# Profitability determining factors of banking sector: Panel data analysis of commercial banks in South Asian countries

**DOI:** 10.3389/fpsyg.2022.1000412

**Published:** 2022-11-10

**Authors:** Deli Yuan, Md. Abu Issa Gazi, Iman Harymawan, Bablu Kumar Dhar, Abu Ishaque Hossain

**Affiliations:** ^1^School of Management, Jiujiang University, Jiujiang, China; ^2^Department of Accounting, Faculty of Economic and Business, Universitas Airlangga, Surabaya, Indonesia; ^3^Mahidol University International College, Mahidol University, Nakhon Pathom, Thailand; ^4^Department of Business Administration, The International University of Scholars, Dhaka, Bangladesh

**Keywords:** OLS model, commercial banks, profitability, panel data, ROA, ROE

## Abstract

**Aim of the article:**

The main purpose of this article was to investigate the impact of the determinants of profitability on the commercial banks in Asian countries. An Asian country like Bangladesh and India was selected as the research field. The present study also pursues to examine the impact of specific factors and macroeconomic factors on the profitability in the Bangladeshi and Indian private commercial banking sectors.

**Methods applied and analysis tools:**

The data were retrieved from the Annual Reports of Indian and Bangladeshi private commercial banks covering the period of 2010–2021. As sample, 40 private commercial banks were considered randomly, of which 20 were from India and 20 were from Bangladesh. The panel data research methodology was used as an estimation technique to analyze the data. Also, the ordinary least squares (OLS) regression model was used to scrutinize data. To check whether the models were appropriate, the Breusch–Pagan Lagrange Multiplier (LM) Test was employed. Banks' specific factors and microeconomic factors showed almost the same variations for both Bangladesh's and India's private banks. All models and tests were evaluated using E-views econometric software.

**The major findings:**

The present study finds that the Return on Asset (ROA) from the banks' specific variables, strength of the Bank size (BS), and Debt to Asset Ratio (DAR) are found to be positive and significant. For banks, the Deposit to Asset Ratio (DTAR) and the Loan to Deposit Ratio (LDR) are found to be negative and significant. The Equity to Asset Ratio (EAR) and Debt to Equity Ratio (DER) do not have any positive/negative impact.

**Contribution, originality, and implications:**

As macroeconomic variables, the inflation rate (IR) and the GDP growth rate (GDPGR) are measured and found to be positive and significant for ROA. As macroeconomic variables, the Inflation Rate (IR) and the GDP Growth Rate (GDPGR) are found to be positive and significant in the case of ROA. The concerned authorities responsible for regulating the financial performance of the banking sector can use the results of this study to take various fruitful decisions on bank profitability.

## Introduction

The importance of the banking industry in ensuring permanent, sustainable, and continuing economic growth cannot be overstated. In Bangladesh and India, the banking system includes both private and public sector banks. Over the decades, the banking industry has undergone major vicissitudes for updating and coping with modern demand for organizing and setting up more banks to cater to the growing needs of the people.

The banking sector, as a proliferating financial institution, plays an active part in a country's economic development (Babu, 2018; Iskandar et al., 2019). Since the recession of 2008, both countries have relied heavily on the banking sector. Prior to the 2008 economic crisis, the improvement in macroeconomic environment and the high level of economic growth were supported by the private banking sector significantly (Andrieş et al., 2016). In fact, the financial sector even provided a map for the expansion and advancement of the world economy. The stability and sustainability of the financial system became a national and international priority as a result of the globalization and financial integration (Javaid, 2016). The stability of a nation's domestic financial system and its capacity to absorb volatility and protect investors were the key factors in determining how the capital moved both domestically and internationally (Hafidh and Burhan, 2021). Commercial banks in Bangladesh needed to be more profit-oriented due to increased domestic and international competition for a stable and adaptable financial system (Sujud, 2020). According to Ali and Puah (2018), nations with a robust and lucrative banking sector were better able to withstand the financial crises, bounce back quickly, and lessen the damage they caused to their economies. The 2008 Global Financial Crises showed the effects of various macroeconomic actions on the banking industry's policy choices and the outcomes of those decisions (Ullah et al., 2020). The problems in the banking system became even more severe as a result of the declining gross domestic product (GDP) and rising inflation (Gazi et al., 2022). Profitability in any company or organization, especially banks, was influenced not only by the internal business environment, but also by the external environment in a variety of ways (Jahan, 2020). Because of the direct and indirect links between the financial crisis and the world economies, there was an impact on every nation.

The banking industry's major mission, among the several financial sectors, has been to convert people's savings into productive investments in a country and thereby to raise the living standards of the people of that country (Rahaman et al., 2020a). Furthermore, after Independence, the banking sectors in both Bangladesh and India have been performing both economic and social operations. As a result, the economies of both countries have started to grow (Gazi et al., 2021a). The socioeconomic situation of Bangladesh and India is almost the same, but Bangladesh is ahead in terms of some economies (Moyo and Tursoy, 2020). The banking sector is one of the main drivers of the economies of both Bangladesh and India (Gazi et al., 2021b). The political and economic conditions of South Asian countries, particularly Bangladesh and India, are completely apart from the fast-growing economies of the world countries (Uddin and Akhter, 2012). The impact of competition on bank profits can lead to policy intervention. As if more profits come from market power, it can negatively affect customers in terms of lower deposit rates, higher loan rates, and qualityless financial services (Yao et al., 2018); alternatively, technical incompetence can also lead to less profit. There is extensive competition among banks for which an appropriate policy response is required. Policymakers must strike a balance between competition and management abilities. Another major factor that might affect profits in both directions is the risk factor (Rahaman et al., 2020b). From decade to decade, the banking sector has been continuing to play an essential role in the country's growth, so it is critical to assess its performance (Lalarukh, 2008). For a financial organization, determining profitability is critical. Banks that want to be profitable should have a good fund management, a lot of capital, and the ability to take risks (Rahayuningsih et al., 2019). Liquidity, capital structure, GDP, assets turnover, size, growth rate, tangibility, tax rate, interest rate, and uniqueness are all factors that have an impact on profitability (Trad et al., 2017).

Previous studies on the profitability determining factors of the banking sector focused on the United States or the European area, with much less discussion on and insight into the banking industry in Asian economies (Soedarmono et al., 2013; Saif-Alyousfi, 2019; Chandra et al., 2020). The present study was conducted to examine the profitability affecting factors of both Indian and Bangladeshi commercial banks. Ordinary least squares (OLS) regression was used to measure the profits of the banking industry in a South Asian representative country like Bangladesh and India. In Bangladesh and India, private, international, state-owned, and Islamic banks collaborate. However, several studies have previously identified those factors that have majorly influenced bank profitability. However, just a few previous research works only have looked into the profitability of banks. As a result, the current study aims to investigate the elements that influence the banking industry's profitability.

## Literature review

This section of the current study looked at a variety of related previously published literature that had studied in depth the factors that influence a bank's profitability using a fixed effects model and panel data approach. Dealing with the period from 2013 to 2017, Islam and Rana (2019) looked at those factors that affected a bank's profitability in Bangladesh's commercial banks. They used the same profit measures for all the three factors: return on asset (ROA), return on equity (ROE), and net interest margin (NIM). While earning variables and asset quality have a substantial positive link with ROA, capital strength does not, according to the researchers. They also discovered that earnings and capital strength have a substantial positive relationship with ROE, with gross domestic product (GDP), interest rate, and inflation rate (IR) having no meaningful impact on the bank profitability. Using the generalized method of moments (GMM) panel data methodology, Horobet et al. (2021) investigated the factors eroding into bank profitability in Central and Eastern European economies (CEE) nations from 2009 to 2018. Inflation rate (IR), budget balance, and non-government spending all had a substantial negative impact, according to the researchers. Credit, lending rates, capitalization percentage, and concentration rate all play a role in deciding a bank's profitability. Using the E-views panel data approach, Sarwar et al. (2018) analyzed 21 commercial banks in Pakistan from 2006 to 2015. They discovered that liquidity, asset management quality, and capital adequacy have a significant influence on a bank's profitability. Sanyaolu et al. (2019) used a fixed effect regression model to examine the factors affecting the bank profitability of Deposit Money Banks in Nigeria from 2008 to 2017. According to their research finding, both inflation and profitability are linked. According to Abdulkabir et al. (2020), the capital structure and operational costs were negatively correlated. El-Kassem (2017) discovered that while variance with an independent variable “Cost to Income Ratio” has a negative and significant impact on the variation in bank performance, variability in the exogenous variable such as “Total Capital Ratio” has a positive and significant impact on a bank's performance. According to Koroleva et al. (2021), size, credit quality, as well as liquidity are internal factors that have a significant positive impact on banks' profitability. State-owned banks are more profitable than other banks because of their larger size, relatively high credit rating, and higher liquidity. On the other hand, the external factor, as expressed by the standardized residuals of GDP, has a detrimental effect on the profitability of banks. The macroeconomic determinants of GDP and inflation rate were discovered to be insignificant by Ngweshemi and Isiksal (2021). According to the empirical findings, bank-specific factors that are under management's direct control provide a better explanation of profitability than macroeconomic factor variables that are out of their direct control. Between 2011 and 2015, Parvin et al. (2019) revealed that the deposit-to-asset ratio (DTAR) was found to have a negative impact on the ROA, but the loan-to-asset ratio and bank size had a positive impact on the bank profitability. In their research, Onofrei et al. (2018) discovered that in the CEE region, which includes seven countries, inflation had no significant positive impact on the bank profitability and that non-governmental credit has a positive impact on the bank profitability. They employed the panel data analysis and analyzed as many as 96 banks. Cetin (2019) studied G20 countries for the period during 2013–2015 using an ordinary least squares (OLS) regression model and stated that there is inflation in developing countries and ROA has a positive relation. Uralov (2020) investigated the factors that influence a bank's profitability in Central European countries from 1996 to 2017 and discovered that the economic growth had a beneficial impact on the bank's ROA. Mahmud et al. (2016) investigated 15 Bangladeshi commercial banks and discovered that the bank's capital had a favorable relationship with the bank profitability. Bank profitability is affected by bank-specific and market-specific factors, according to Hossain and Khalid (2018), Odusanya et al. (2018), Mosharrafa and Islam (2021) looked at bank's profitability in Bangladesh from 2007 to 2017 and discovered a favorable relationship between loan and efficient management and bank profitability. Menicucci and Paolucci (2016) examined, from 2006 to 2015, the profitability and endogenous variables of the 28 biggest banks in the European Union (EU). A significant link between profitability as well as liquidity ratio (LR), bank size, and deposit ratio is supported by empirical findings. Profitability, meanwhile, is negatively impacted by the asset quality based on the results of the regression. Additionally, Abel and Le Roux (2016) used fixed effect model panel regression models to conduct an empirical study of Zimbabwe's banking sector between 2009 and 2014. They discovered that there is empirical support for the positive relationships between the size of liquid funds, capital volume, total assets, and cost efficiency and profitability. To determine the degree of impact on the financial performance over the 10-year period from 2006 to 2016, Ozgur and Gorus (2016) conducted research on the deposit banks' profitability of Turkey using the OLS method. The study's findings indicated that the capital size, asset quality, the ratio of interest earnings to total assets, and the interest rate set by the central bank all have an impact on a bank's profitability. The study also showed how the Turkish banks' profitability was negatively impacted by the Global Financial Crisis of 2008. Using the panel data regression analysis, Mehta and Bhavani (2017) conducted research on the variables that influenced the profitability of 19 commercial banks operating in the United Arab Emirates (UAE) between 2006 and 2013. They discovered three elements, including (i) cost-effectiveness, (ii) capital ratio, and (iii) asset quality that significantly improve the bank's profitability. Phan et al. (2020) studied 10 Vietnamese-listed commercial banks, which were investigated between 2008 and 2018 using the instrumental variables (IV) regression and OLS regression model. They discovered that factors like state ownership, loan size, loan to GDP ratio, inflation rate (IR), and operating efficiency all have a positive effect on the profitability. Gwachha (2019) has examined the macroeconomic and bank-specific factors that affected the Nepalese banking sector's profitability from 2004 to 2013. He came to the conclusion that the loan portfolio has a significant negative impact on the profitability of the bank. Hosen (2020) investigated 23 commercial banks in Bangladesh and discovered that interest rate spread, capital adequacy, and liquidity are all important factors. Lestaria et al. (2021) discovered that liquidity and leverage have a negative impact on the profitability (ROE), whereas the bank size has a positive impact. Iskandar et al. (2019) investigated Malaysia's banking sector and found that various factors such as managerial efficiency, liquidity risk, and credit risk all have a role to play in the bank's profitability. This also includes other factors such as earnings variable, asset structure, capital strength, and liquidity. According to Imtiaz et al. (2019), Gazi et al. (2021b) have a considerable favorable impact on profitability. Noman et al. (2015) and Ullah et al. (2016) discovered a positive link between bank profitability and economic growth. They also discovered that real interest rates had a negative impact on profitability, but inflation, size, and capital sufficiency all have a favorable impact. Hasanov et al. (2009) and Syathiri et al. (2020) discovered that the size of a bank, the number of loans it makes, and the amount of money it lends out are all factors. Bank profitability is impacted by the adequacy of financial assets, the deposit-to-GDP level, and the operating efficiency, according to Brahmaiah (2018). Mohanty and Krishnankutty (2018) looked into the profitability of Indian banks. They discovered that capital creditworthiness and sufficiency ratios have a positive link with profitability, whereas size, loan-to-deposit ratio (LDR), expense ratio, and GDP growth all have a negative correlation. Huge capital, according to Chowdhury (2015) and Ozili (2016) is linked with banks' desire to enhance profits. Hu and Xie (2016) and Tan et al. (2016) calculated that taking risks has a positive impact on bank profitability and that debt risk has a negative impact on bank profitability. In the context of Indonesia, Hasan et al. (2020) investigated the impact of banks' profitability in regard to the two variables ROA and ROE. They discovered that a number of factors, including the return on equity (ROE), net interest margin, capital ratio, and liquidity, have a big impact on a bank's profitability. Using a generalized method of moments (GMM) system, Jonathan and Xuan (2019) carried out a study on Nigeria and demonstrated how cost-efficiency plays a significant role in achieving profitability in developing nations. Belke and Unal (2017) used the Generalized Method of Moments (GMM) to conduct their study in Azerbaijan, which has an oil-dependent economy, and found a positive relationship between profitability and internal and external factors like total assets, asset, and liability, price of oil, rate of inflation, economic cycle, etc. A study on the variables influencing the profitability of 27 banking sectors in Asian countries from the years 2012 to 2016 was conducted by Dao and Nguyen (2020). The most striking similarity between all entities is that they all recorded the significantly inverse correlation between operational risk and banking profitability. In particular, ROA and ROE are defined as profitability indicators. Similar to how Malaysia's model shows no significant impact, Vietnam's and Thailand's models show a significantly negative impact of bank size on the profitability. Ugo et al. (2016) studied the profitability of the European Union (EU) banks from 2001 to 2018 and discovered a positive association between profitability and GDP growing rate. Opoku et al. (2016) investigated the factors that influence bank profitability in Ghana. Non-performing loans (NPLs) have a detrimental impact on the bank profitability, according to their research. Profitability and economic growth have a favorable association, according to Alhassan et al. (2016). Alzoubi (2018) investigated the profitability of Jordanian banks. According to him, bank size (BS), stocks to assets, and deposits have a greater influence on the traditional bank's profitability. Bhattarai (2018) showed that the capital adequacy ratio (CAR) and the annual inflation rate are related. Islam and Nishiyama (2016) studied banks' profitability of South Asian countries using GMM methods for analyzing panel data. They observed a positive association between ROA and interest rate. According to Nisar (2015), a significant number of non-performing loans (NPLs) reduce the bank profitability. Independent auditors, according to Alina (2015), have a major impact on bank profitability. Non-performing loans (NPLs) and interest rates, according to Ariyadasa et al. (2016), have a detrimental impact on bank profitability. In Syria, Al-Jafari and Alchami (2014) employed the GMM approach to investigate the deciding factors of bank profitability. Inflation and the rate of economic development, they discovered, have a favorable impact on profitability. Interest rates have a considerable influence on bank profitability, according to Aydemir and Ovenc (2016). According to Saeed (2014), Regehr and Sengupta (2016), inflation has an adverse impact on bank's profitability, while bank size (BS) has an advantageous impact. According to Isah (2018), Tabash et al. (2019), and Islam and Bhhuiyan (2021), profitability is positively impacted by the bank size (BS), but negatively impacted by overhead expenses, liquidity, and non-performing loan (NPL).

Bank profitability assessors are chosen, based on the study's nature and goal. Internal components are referred to as bank-specific variables, and independent variables include size of the bank, debt to asset ratio (DAR), deposit to asset ratio (DTAR), equity to asset ratio (EAR), debt to equity ratio (DER), and loan to deposit ratio (LDR) which are all factors to consider. This study looked at two macroeconomic parameters, the GDP growth rate (GDPGR) and the inflation rate (IR), which are referred to as bank external factors.

In the previous literature, we have noticed that the profit of a bank depends on some external and internal factors. Comparatively internal factors are more important and effective toward banks' profits that are proved in previous empirical studies. Most of the previous researches have profound insights into these issues and have quantified that Assets Quality, Bank's size (Iannotta et al., 2007; Moinescu, 2008), Capital ratio (Athanasoglou et al., 2006), and Management competence are the greatest significant determinants of a bank's profitability. Pre- and post-crisis researches using the same bases to analyze bank profitability (Athanasoglou et al., 2006; Brei et al., 2020; Budhathoki et al., 2020; López et al., 2020; Tercero-Lucas, 2020) showed similar results. The global financial crisis has had a favorable impact on bank profitability (Abdullah and Tan, 2017).

This research addresses a gap in the literature by examining the impact of bank-specific and macroeconomic factors on bank profitability in Bangladesh and India. Researchers frequently examined banking and financial markets drivers, which were found to have a greater impact on banks' profit ratios than macroeconomic factors. We propose the following hypothesis based on a survey of the literature.

*H1: Banks' specific factors/variables (internal factors) have no significant impact on profitability (ROA ad ROE) of banking sector in Bangladesh and India*.
*H2: Microeconomic factors/variables (external factors) have no significant impact on profitability (ROA ad ROE) of banking sector in Bangladesh and India*.
*H3: There is no significant difference between Bangladesh and India's banking sector based on Profitability impacting factors*.

## Research methods and procedures

### Sample selection

To select the sample banks for the present study, two lists of total banks were collected from Bangladesh and India. Bangladesh and India have been selected purposively from among the South Asian countries. The present study comprises of 43 private commercial banks from Bangladesh (Moyo and Tursoy, 2020) and 89 commercial banks from India as per the population. Based on the data availability, 40 commercial banks were chosen at random from the total population, with 20 banks from Bangladesh and 20 from India. This study employed secondary data from the last 12 years (2010–2021), and 300 observations were created using the panel data approach.

### Model

The profitability determining factors of the banking sector of Bangladesh and India are estimated by the following models;

***Model-1:*** ROA _it_ = α_i_ + β_1_(BS) _it_+β_2_(DAR)_it_+β_3_(DTAR)_it_+β_4_(EAR)_it_+β_5_(DER)_it_+β_6_(LDR)_it_+β_7_(IR)_it_+β_8_(GDPGR)_it_+*u*_it_

**Model-2:** ROE _it_ = α_i_ + β_1_(BS) _it_+β_2_(DAR)_it_+β_3_(DTAR)_it_+β_4_(EAR)_it_+β_5_(DER)_it_+β_6_(LDR)_it_+β_7_(IR)_it_+β_8_(GDPGR)_it_+*u*_it_

Where, α = Intercept of the model; i = Index of Banks; t = Time index; β_*k*_ = Regression Coefficient to be estimated; *u*_it_ = Random error term.

ROA, Return on Asset; ROE, Return on Equity; DTAR, Deposit to Asset Ratio; BS, Bank Size; DER, Debt to Equity Ratio; LDR, Loan to Deposit Ratio; DAR, Debt to Asset Ratio; EAR, Equity to Asset Ratio; IR, Inflation Rate (IR) and GDPGR, GDP Growth Rate.

### Model assumptions

Some assumptions used it to deduce the OLS estimators in linear regression models, and these are as follows (Kennedy, 2008);

Assumption 1: A random sample of observations is used. The regression model is linear in the coefficients and error term.


$$\begin{array}{l} Y=\;\beta_{0}+\beta_{1}X_{1}+\beta_{2}X_{2}{+}^{\ldots \ldots ..}+\;\beta_{k}X_{k}+u \end{array}$$

Assumption 2: The conditional mean should be zero.


$$\begin{array}{l} E\left(u/X\right)=\;0 \end{array}$$

The error term accounts for variation in the dependent variable that is not explained by the independent variables. The error term's values should be determined by chance.

Assumption 3: There is no multicollinearity or perfect collinearity.

Because there is only one independent variable in a simple linear regression model, this assumption will hold true by default. However, there is more than one independent variable in multiple linear regression models. The OLS assumption of no multicollinearity states that the independent variables should not have a linear relationship.

Assumption 4: There is no autocorrelation and there is homoscedasticity.


$$\begin{array}{l} Var\;\left(u/X\right)=\;\sigma^{2}\\ Cov\left(u_{i}u_{j}/X\right)=0\;for\;i\neq j \end{array}$$

If the variance is not constant dependent on X's, the linear regression model has heteroscedastic errors which are likely to produce incorrect estimates. According to the no autocorrelation OLS assumption, different observations' error terms should not be correlated with one another.

Assumption 5: The error term has a normal distribution.

OLS does not need the error terms to have a normal distribution for producing unbiased estimates with the least variance. Satisfying this assumption, on the other hand, it becomes important when some additional finite-sample properties must be defined. It should be noted only the error terms must be normally distributed. It is not necessary for the dependent variable Y to be normally distributed.

### Outlier test

Statistically, an outlier is a value that lies abnormally far from other values within a population. Outliers are observations with unusual values for a single variable. A non-random distribution of outliers can reduce normality. Statistical tests lose power as a result of this increase in error variance. Therefore, we tested outliers before estimating the model. As a result of the outlier detections, errors were reduced and the generalization ability of the results was enhanced. A total of 300 observations were selected after checking 12 outliers from 312 observations.

### Data analysis instruments

E-views software is cast off to evaluate all models and investigations as panel data analysis tools. The Random-Effect Model, Fixed Effect Model, and Pooled Ordinary Least Square Model were employed in this investigation (OLS). Error term, intercept, and coefficient regression are examples of specific assumptions (Kennedy, 2008). The Hausman experiment was utilized in this study to compare the results of the fixed effect and random effect specifications. The BPLM–Breusch–Pagan Lagrange Multiplier Test is also used to ensure that the models are suitable. However, the aforementioned regression models have been presented in this regard. The panel data regression model was used to look at specific aspects of banks as well as macroeconomic issues that influence profitability.

### Variables

#### Dependent variables

The purpose of this study was to investigate the impact of profit determining aspects on the banking industry in Bangladesh and India on governing bank's specific variables and macroeconomic variables. According to Isayas (2022), there are two indicators of profitability of the banking industry that are returns on assets (ROA) and return on equity (ROE) which are considered dependent variables in this study (Table 1).

**Table 1:** Description of dependent variables.

**Variables**	**Measurement**	**Source of data collection**	**References**
**Profitability indicators**			
Return on asset	ROA% = Net profit after tax/total assets	Bank scope	Athanasoglou et al., 2008; Masood and Ashraf, 2012; Francis, 2013; Perera et al., 2013; Noman et al., 2015
Return on equity	ROE% = Net profit after tax/ total equity	Bank scope	

Source: Authors' own explanations.

**Return on Asset (ROA):** ROA is the profit ratio that shows the bank's ability to make a profit over their whole assets affianced in the banking industry. ROA is the widely used and key ratio of gauging bank's profitability (Tan, 2016). This ratio supports banks to understand the capability to invest and use financial resources to make a profit.

**Return on Equity (ROE):** ROE refers to the return of shareholders to their equity. Return on Equity (ROE) flouts the risks allied with financial leverage and height leverage is habitually resolute by regulations. It denotes a bank's profit generated by the amount invested by its shareholders (Olorunniwo and Hsu, 2006). ROE is important to calculate to see how the bank management is using shareholder funds. ROE assists quantity a bank's ability to use investment funds to increase earnings.

#### Independent variables

The present study considered the determining factors of the profitability of banks, which are categorized into two i) Banks' specific factors/variables (internal factors) and ii) industry and microeconomic factors/variables (external factors). We use the following internal factors (Banks' specific factors/variables); BS, DAR, DTAR, EAR, DER, and LDR. Microeconomic determinants comprised GDP Growth Rate (GDPGR) and Inflation Rate (IR). Some selected independent variables were utilized to investigate the causes affecting Bangladesh and India's banking sectors' profitability (Table 2).

**Table 2:** Description of independent variables.

**Variables**	**Measurement**	**References**
**Banks' specific factors/variables (internal factors)**		
Date-to-asset ratio	DAR% = Total liabilities/Total assets	Gazi et al., 2021a
Equity-to-asset ratio	EAR% = Total equity/Total assets	Staikouras and Wood, 2004
Loan-to-deposit ratio	LDR% = Total loans/Total deposits	Menicucci and Paolucci, 2016; Regehr and Sengupta, 2016
Deposit-to-asset ratio	DTAR% = Total deposit/Total assets	Rahaman and Akhter, 2015
Debt-to-equity ratio	DER% = Total Liabilities/Total equity	Pradhan and Khadka, 2017
Bank size	BS= {ln(Total Bank Assets)}	Petria et al., 2015
**Microeconomic factors/variables (external factors)**		
GDP growth rate	GDPGR% = (GDP_n_-GDP_n − 1_/GDP _n − 1_) Yearly percentage change in the gross domestic Product	Olorunniwo and Hsu, 2006; Al-Jafari and Alchami, 2014; Aydemir and Ovenc, 2016; Ugo et al., 2016
Inflation rate	IR = Inflation growth rate	

Source: Authors' own explanations.

### Houseman test, Brush–Pagan Lagrange multiplier, and panel data unit root test

Houseman test, Brush–Pagan Lagrange multiplier (LM), and F-test were used to select the appropriate model for this study. The F-test is selected by focusing on Table 3 first to indicate between the pulled and fixed effect models.

**Table 3:** Houseman test, Brush–Pagan Lagrange multiplier (LM), and Panel data unit root test, and result of F-test.

		**Overall**		**Bangladesh**		**India**	
**Tests**		**Model-1**	**Model-2**	**Model-1**	**Model-2**	**Model-1**	**Model-2**
F Test	F Statistic	2.498	3.249	2.611	4.012	3.012	4.075
	*P*-value	0.011	0.021	0.009	0.005	0.009	0.0014
Breusch-Pagan Test	Chi-Square statistic	4.768	10.588	3.999	11.247	5.587	10.8576
	*P*-Value	0.031	0.000	0.031	0.000	0.029	0.000
Hausman Test	Chi-Square statistic	1.821	7.215	1.915	6.345	1.758	7.1857
	*P*-Value	0.941	0.404	0.956	0.340	0.889	0.399
*H_0_ = The pooled model is better than the fixed effect model, H_1_ = The fixed effect model better than the pooled model*							
*H_0_ = The random effect model is better than the fixed effect model, H_1_ = The fixed effect model is better than the random effect model*							
**Panel data unit root test (Levin, Lin and Chu)**		
**Variables**	**t-statistics**	***p*** **-value**
ROA	−10.42512	0.000
ROE	−9.02145	0.000
BS	−6.08754	0.000
DAR	−7.85214	0.000
DTAR	−9.98745	0.000
EAR	−12.12441	0.000
DER	−7.45872	0.000
LDR	−12.45879	0.000
IR	−6.95123	0.000
GDPGR	−13.75315	0.000
*H_0_ = Existence of a common unit root, H_1_ = Absent of a common unit root*.		

Table 3 indicates that the LM testing was applied to select the appropriate model between random and combined effects. The results revealed that overall random effect models were pooled at a significant 5% level of chi-square quality for Model 1 and at 1% for Model 2. The fixed and random effects are not accepted pooled model base on LM test and F test. Individually the effects for Bangladesh and India also remain the same and the rejected pooled model at significant 5 and 1% levels is based on the F-test and LM test. Breusch–Pagan Test and Hausman Test were conducted to select the model between static and random effects for overall and Bangladesh, and India. At the level of 5%, significant *p* value and chi-square statistics are very high in the case of both models for random effect. Therefore, the random effect model is pertinently used for both models for ROA and ROE.

## Analysis and results

Table 4 presents the descriptive statistical value for all key variables and shows the affirmative mean values of all independent variables. The value is used for measuring banks' performance of both Bangladesh and Indian banks overall. The independent variables ROA and ROE have mean values of 0.0995 and 0.127, whereas the median values are 0.0107 and 0.138 (SD = 0.0172 and 0.326), respectively.

**Table 4:** Descriptive Statistics for both Bangladesh and India (overall).

**Variables**	**Observation**	**Mean**	**Median**	**SD**	**Sample variance**	**Maximum**	**Minimum**
ROA	300	0.0995	0.0107	0.0172	0.032	0.048	1.25
ROE	300	0.127	0.138	0.326	0.121	2.032	5.12
BS	300	24.7	26.9	0.791	0.68	3.254	9.875
DAR	300	23.75	24.45	0.889	16.28	7.855	48.258
DTAR	300	0.852	0.831	0.492	0.421	2.587	6.846
EAR	300	0.641	0.759	0.763	0.087	1.875	3.357
DER	300	13.4	12.8	9.26	6.26	8.214	14.258
LDR	300	0.947	0.857	0.188	3.034	2.680	6.279
IR	300	3.985	2.844	1.055	0.069	6.578	7.057
GDPGR	300	2.061	1.0788	0.149	0.081	4.890	6.014

The mean and median value for bank size (BS) are 24.7 and 26.9%. Deposit to asset ratio (DAR) has a mean value of 23.75%. The table also shows that the mean value of GDPGR is 2.061%, whereas for the inflation rate (IR) it is 3.985. DER, BS, and DAR have slightly higher mean and median value. The results of Table 4 suggest that ROE and ROA are 0.10 and 0.107% (mean value) separately, which indicates that the performance of Bangladeshi banks and Indian banks is not good enough based on profitability.

From Table 5, we can conclude the results of the above table that there is no significant concern as regards multicollinearity based on Table 4 because independent variables do not have high correlations and the variance inflation factor (VIF) for all predictor variables is less than 5.

**Table 5:** Pearson correlation matrix and VIF.

**Variables**	**ROA**	**ROE**	**BS**	**DAR**	**DTAR**	**EAR**	**DER**	**LDR**	**IR**	**GDPGR**
ROA	1									
ROE	−0.182	1								
BS	−0.147*	0.725	1							
DAR	−0.091	−0.568**	0.051	1						
DTAR	0.736**	0.318	0.122	0.082*	1					
EAR	0.075*	0.182**	−0.035*	−0.321	0.510**	1				
DER	−0.082	0.256*	−0.134*	0.514**	0.0545	−0.058*	1			
LDR	0.921**	−0.552*	−0.311*	0.601	0.349	−0.015	0.096**	1		
IR	0.309	0.0741*	−0.056*	−0.014	0.211**	−0.302	−0.572*	0.089*	1	
GDPGR	0.231*	0.682**	−0.079	0.164**	0.381	0.182*	0.479*	−0.1215	0.387	1
VIF	**2.452**	**1.097**	**3.547**	**2.782**	**1.087**	**2.273**	**1.873**	**2.743**	**1.089**	**2.821**

^*^P-value < 0.05, ^**^P-value < 0.01.

Table 6 shows the descriptive statistics of profitability indicators of the overall performance of the banking sector of both Bangladesh and India, and also individually. As a prominent financial institution, the banking sector of Bangladesh and India is not in a favorable position as a whole. Both countries could not ensure a good position. As a recognized profitability indicator, ROA and ROE are not looking sound. The Bangladesh banks showed more volatility than Indian banks. ROA showed almost the same variations for Bangladesh and India's private banks. But ROE showed wide variations (14.06%) for the Indian private banks. For Bangladeshi private banks, ROA is 0.79% and ROE is 0.98%, which indicates the miserable condition; on the other hand for India's private banks, ROE values are 1.14 and 13.08%, which indicate although it is not a much better position, but India is in a better position than Bangladesh.

**Table 6:** Profitability indicators' summary.

**Variables**	**Observation**	**Mean**	**Median**	**SD**	**Sample variance**	**Maximum**	**Minimum**
**Overall private commercial banks**
ROA	300	0.0995	0.0107	0.0172	0.032	0.048	1.25
ROE	300	0.127	0.138	0.326	0.121	2.032	5.12
**Private commercial banks of Bangladesh**
ROA	180	0.79	0.980	0.38	0.0.18	0.52	2.40
ROE	180	6.12	0.185	4.25	7.64	7.11	14.28
**Private commercial banks of India**
ROA	120	1.14	1.03	0.48	0.15	0.41	2.27
ROE	120	13.08	14.06	3.07	10.15	7.55	19.33

Table 7 shows the brief descriptive statistics of independent variables. The overall performance of the private banking sector of Bangladesh and India in response to independent variables is not bad but not so good either. Among the banks' specific factors/variables (internal factors), some position is good. The mean values of the bank size (BS) are 24.7%, deposit to asset ratio 23.75%, and debt to equity ratio is 13.4% which indicate the positive impact on the profitability of banks. The mean values of DTAR, EAR, and LDR are slightly lower. On the other hand, as microeconomic factors/variables (external factors) the inflation rate (IR) and GDP growth rate (GDPGR) indicate a comparatively strong position. The mean and median value of IR are 3.985 and 2.844%, and the mean and median value of GDP growth rate are 2.061 and 1.0788%, respectively. Banks' specific factors and microeconomic factors showed almost same variations for Bangladesh and India's private banks.

**Table 7:** Summary of the independent variables.

**Variables**	**Observation**	**Mean**	**Median**	**SD**	**Sample variance**	**Maximum**	**Minimum**
**Overall private commercial banks**							
BS	300	24.7	26.9	0.791	0.68	25.45	65.04
DAR	300	23.75	24.45	0.889	16.28	38.47	52.142
DTAR	300	0.852	0.831	0.492	0.421	0.075	0.871
EAR	300	0.641	0.759	0.763	0.087	0.08	0.255
DER	300	13.4	12.8	9.26	6.26	5.12	14.252
LDR	300	0.947	0.857	0.188	3.034	1.65	3.252
IR	300	3.985	2.844	1.055	0.069	3.214	4.221
GDPGR	300	2.061	1.0788	0.149	0.081	5.147	15.547
**Private commercial banks of Bangladesh**							
BS	180	16.22	22.71	0.55	0.32	6.88	10.25
DAR	180	49.58	58.88	7.77	48.78	65.96	82.25
DTAR	180	4.32	3.57	1.85	1.06	1.98	6.05
EAR	180	1.98	0.08	0.48	0.32	1.55	3.12
DER	180	8.50	7.98	0.69	0.55	5.82	11.02
LDR	180	4.92	6.72	0.90	0.77	3.60	7.80
IR	180	7.87	7.90	2.80	5.73	4.78	11.89
GDPGR	180	7.52	6.99	1.78	5.25	4.65	11.02
**Private commercial banks of India**							
BS	120	17.23	7.34	0.43	0.19	5.99	8.12
DAR	120	76.18	75.88	6.99	44.45	55.85	90.02
DTAR	120	3.12	2.44	1.05	1.03	1.87	5.55
EAR	120	1.89	0.09	0.48	0.24	1.2	3.87
DER	120	7.77	7.55	0.70	0.50	6.01	10.08
LDR	120	5.61	5.79	0.94	0.88	3.20	8.00
IR	120	8.52	8.35	2.40	5.66	4.50	12.01
GDPGR	120	7.58	7.55	1.82	3.21	3.91	11.30

ROA, Return on Asset; ROE, Return on Equity; DTAR, Deposit to Asset Ratio; BS, Bank Size; DER, Debt to Equity Ratio; LDR, Loan to Deposit Ratio; DAR, Debt to Asset Ratio; EAR, Equity to Asset Ratio; IR, Inflation Rate (IR) and GDPGR, GDP Growth Rate.

Furthermore, a Table 7 result reveals that throughout the study period, the Bangladeshi and Indian private banks look for more strength. But some internal factors are strong for Bangladesh and some factors are strong for India. The ratio of bank size (BS) (76.18%), DAR (17.23%), and LDR (5.61%) of Indian private banks is higher than those of the Bangladeshi private banks. This indicates that the Indian private banks managed their operational activities efficiently. The ratio of EAR (1.98%) and LDR (8.50%) of Bangladeshi private banks is higher than that of the Indian private banks. The private banks of Bangladesh and India appear to be sturdier by devouring a fairly high ratio of GDP growth rate (GDPGR) and that are 7.52 and 7.58%, respectively. The inflation rate is almost the same for both countries (7.87 and 8.52%).

Table 8 presents the Pearson's correlation coefficients of the independent variables in relation to dependent variables such as ROA and ROE used in this study. The Pearson's correlation coefficients are depicted below the diagonal. When the coefficient of correlation between variables exceeds 0.80 (Alharbi, 2017) then the multicollinearity problems occur. The matrix shows that in general the correlation between the banks' specific factors/variables (internal factors) is not strong, suggesting that multicollinearity is not austere or non-existent.

**Table 8:** Correlation coefficients based on 300 observations.

**Variables**	**ROA**	**ROE**	**BS**	**DAR**	**DTAR**	**EAR**	**DER**	**LDR**	**IR**	**GDPGR**
ROA	1.0000	0.4256	0.1588	0.0896	−0.0000	0.5296	0.0787	0.0533	0.8750	−0.1720
ROE		1.0000	−0.1215	−0.568	−0.0094	0.0565	−0.3193	0.1313	−0.4325	−0.1873
BS			1.0000	0.4565	−0.1235	0.3370	0.4111	−0.0342	0.3829	−0.0342
DAR				1.0000	0.1872	0.2584	0.6210	−0.1247	0.1752	0.7698
DTAR					1.0000	0.3580	−0.0988	−0.3026	0.2542	0.0872
EAR						1.0000	0.1769	−0.0663	−0.5471	−0.0322
DER							1.0000	−0.0628	0.0746	0.0816
LDR								1.0000	0.6541	0.2029
IR									1.0000	0.1584
GDPGR										1.0000

ROA, Return on Asset; ROE, Return on Equity; DTAR, Deposit to Asset Ratio; BS, Bank Size; DER, Debt to Equity Ratio; LDR, Loan to Deposit Ratio; DAR, Debt to Asset Ratio; EAR, Equity to Asset Ratio; IR, Inflation Rate (IR) and GDPGR, GDP Growth Rate.

The linearity difficulties in the explanatory variables are not apparent in the correlation matrix (does not exceed 0.80). ROE and BS, DAR, EAR, DER, and IR are favorably associated with ROA, however GDPGR is adversely connected with ROA, as seen in the matrix. Finally, ROE is positively associated with EAR, LDR, and ROA, whereas it is adversely associated with BS, DAR, DTAR, DER, IR, and GDPGR.

Table 9 depicts the impact of macroeconomic variables and firm-specific on bank profitability, as measured by ROA and ROE. The following are the places where regression results can be found:

**Table 9:** The results of the random effects estimation as measured by ROA and ROE for bank profitability.

	**Dependent variable: ROA (Overall)**				**Dependent variable: ROE (Overall)**			
**Variable**	**Model-I**		**Model-II**		**Model-I**		**Model-II**	
	**Coefficient**	***P-*value**	**Coefficient**	***P-*value**	**Coefficient**	***P-*value**	**Coefficient**	***P-*value**
C	0.028	0.316	0.033	0.003	0.096	0.740	0.610	0.004
BS	0.012	0.918***	0.215	0.854***	0.022	0.310***	0.254	0.774***
DAR	0.069	0.000***	0.0691	0.000***	0.152	0.252	0.872	0.587
DTAR	−0.372	0.024**	−0.334	0.010***	−7.324	0.003***	−7.992	0.002***
EAR	0.005	0.252	0.365	0.0258	0.277	0.013**	0.221	0.01 ***
DER	−5 05	0.578	0.854	0.954	−0.009	0.000***	−0.009	0.000***
LDR	−0.075	0.019***	−0.082	0.001***	0.003	0.992	0.875	0.158
IR	0.875	0.125***	0.596	0.000**	−0.082	0.789**	−0.654	0.100**
GDPGR	0.258	0.0100**	0.364	0.010***	0.658	0.852***	0.753	0.854***
Observations	300		300		300		300	
Adjusted R–squared	0.338		0.342		0.218		0.132	
F–statistic	39.174		63.852		10.985		19.385	
Prob (F-statistic)	0.000***		0.000***		0.000***		0.000***	
Durbin-Watson	1.939		1.908		1.986		1.955	

^**^ *p*<*5%*, ^***^ *p* < 1%. ROA, Return on Asset; ROE, Return on Equity; DTAR, Deposit to Asset Ratio; BS, Bank Size; DER, Debt to Equity Ratio; LDR, Loan to Deposit Ratio; DAR, Debt to Asset Ratio; EAR, Equity to Asset Ratio; IR, Inflation Rate (IR) and GDPGR, GDP Growth Rate.

The result of pooled OLS estimates equations considering ROE and ROA as dependent variables of this study. Table 9 shows that the models are well fitted with data moderately and are having the adjusted R-square of 0.338 for ROA and 0.218 for ROE (model-1) and 0.342 for ROA and 0.132 for ROE (model-2). The coefficient values of all independent variables are statistically significant in F-statistics at the 1% significance level (Field, 2009). The study reveals that the strength of Bank size (BS) and Debt to Asset Ratio (DAR) is positive and significant in explaining ROA from the banks' particular characteristics. The Loan to Deposit Ratio (LDR) is also shown to be negative and important when it comes to a bank's Deposit to Asset Ratio (DTAR). Neither the Equity to Asset Ratio (EAR) nor the Debt to Equity Ratio (DER) has any positive or negative implications. If the macroeconomic variables such as ROA, IR, and GDPGR are positive and significant, bank-specific variables, such as Bank size (BS) and Equity to Asset Ratio (EAR) are positive and significant, whereas the Deposit to Asset Ratio (DTAR) and Debt to Equity Ratio (DER) are negative and insignificant (Iannotta et al., 2007). In the case of ROE, the GDP growth rate is to be considered positive significant, whereas the inflation rate (IR) is to be found negative significant. Furthermore, the DW (Durbin–Watson) test values for models (I) and (II) are 1.939, 1.908, 1.986, and 1.955, respectively, implying that there is no autocorrelation grounded on the decree of thumb (Hendry, 2000). Following that, two explanatory variables in this model (EAR and DER) are not significant for ROA and DAR, while LDR is not significant for ROE (Abdulkabir et al., 2020; Horobet et al., 2021), thus we may delete these variables from the model and reguess the model using GSA (General Specific Approach) (Sukmana and Febriyati, 2016) to get the most efficient model.

Table 10 displays the regression results for Bangladesh, which show the impact of firm-specific and macroeconomic variables on bank profitability, as dignified by ROA and ROE as dependent variables. The results of this model determine that Bank size (BS), to Asset Loan to Deposit Ratio (LDR), and Debt to Asset Ratio (DAR) are found to have positive significant impact on ROA, whereas the Deposit to Asset Ratio (DTAR) was found to have a negative impact as banks' specific variables. Bank's macroeconomic variable GDP growth rate has a positive impact on ROA. For ROE, Debt to Asset Ratio (DAR) and Deposit to Asset Ratio (DTAR) have positive significant impact on ROE, but Bank size (BS), Loan to Deposit Ratio (LDR) are found to have negative significant impact on ROE as banks' specific variables. Whereas macroeconomic factors like GDP growth rate and Inflation rate (IR) were found to have a negative impact on ROE. The Coefficient standards of the all independent variables are conjointly statistically significant of F-statistics at the significant level of 1% and adjusted R-square of 0.437 for ROA and 0.0332 for ROE which proved the models are well fitted for the Bangladeshi private commercial banking sector.

**Table 10:** Bangladesh's bank profitability regression results using Random Effects Estimation (measured by ROA and ROE).

**Dependent variable: ROA**			**Dependent variable: ROE**		
**ROA analysis**	**Coefficient**	***P*-value**	**ROE analysis**	**Coefficient**	***P*-value**
C	0.023	0.315	C	0.0345	0.0003
BS	0.011	0.010**	BS	−0.0258	0.001**
DAR	0.071	0.000***	DAR	0.0709	0.000***
DTAR	−0.383	0.025**	DTAR	0.349	0.01***
EAR	0.005	0.338	EAR	1.258	0.065
DER	−4.024	0.688	DER	5.125	0.593
LDR	0.389	0.001***	LDR	−0.483	0.001***
IR	0.398	0.355	IR	−0.0347	0.031**
GDPGR	0.025	0.029**	GDPGR	−0.842	0.028***
Observations	180		180		
Adjusted R-squared	0.437		0.332		
F-statistic	28.587		54.753		
Prob (F-statistic)	0.000***		0.000***		
Durbin-Watson	1.899		1.901		

^**^ *p* < 5%, ^***^ *p* < 1%. ROA, Return on Asset; ROE, Return on Equity; DTAR, Deposit to Asset Ratio; BS, Bank Size; DER, Debt to Equity Ratio; LDR, Loan to Deposit Ratio; DAR, Debt to Asset Ratio; EAR, Equity to Asset Ratio; IR, Inflation Rate (IR) and GDPGR, GDP Growth Rate.

Table 11 demonstrates that the models are relatively well matched with data, with adjusted R-squares of 0.872 for ROA and 0.758 for ROE. All of the independent variables' coefficient values are statistically significant in F-statistics at the 1% significance level. The Indian private commercial banking sector appears to be strongly based on the banks' unique ROA characteristics. We discovered that the debt to asset ratio (DAR), the deposit to asset ratio (DTAR), and the debt to equity ratio (DER) are all positive and significant when it comes to ROA. The equity to asset ratio (EAR) of banks is negative and considerable. GDP growth rate (GDPGR) and inflation rate (IR) are both negative and major macroeconomic determinants on ROA. Furthermore, Table 11 shows those banks' unique variables, such as equity to asset ratio (EAR) and loan to deposit ratio (LDR), are positive and substantial, but debt to asset ratio (DAR) and deposit to asset ratio (DTAR) have a negative and significant impact on ROE. Banking growth in terms of bank deposit to GDP growth rate has a negative significant impact on ROE in macroeconomic variables. There is no autocorrelation based on the Durbin–Watson (DW) test value.

**Table 11:** Random effects estimation regression for banks' profitability in India (measured by ROA and ROE).

**Dependent variable: ROA**			**Dependent variable: ROE**		
**ROA analysis**	**Coefficient**	***P*-value**	**ROE analysis**	**Coefficient**	***P*-value**
C	1.480	1.521	C	38.70	0.028***
BS	−0.811	0.788	BS	−1.022	0.398
DAR	4.851	0.022***	DAR	−1.066	−0.022**
DTAR	4.083	0.001**	DTAR	−0.844	−0.00***
EAR	−2.321	−0.010***	EAR	3.221	0.002***
DER	2.344	0.023**	DER	1.855	1.817
LDR	−0.047	0.000	LDR	5.185	0.05***
IR	−2.99	−0.041**	IR	1.988	0.026**
GDPGR	−2.011	0.033**	GDPGR	−0.591	−0.165
Observations	120		120		
Adjusted R-squared	0.872		0.758		
F-statistic	49.0257		54.0874		
Prob (F-statistic)	0.000***		0.000***		
Durbin-Watson	1.785		1.901		

^**^ *p* < 5%, ^***^ *p* < 1%. ROA, Return on Asset; ROE, Return on Equity; DTAR, Deposit to Asset Ratio; BS, Bank Size; DER, Debt to Equity Ratio; LDR, Loan to Deposit Ratio; DAR, Debt to Asset Ratio; EAR, Equity to Asset Ratio; IR, Inflation Rate (IR) and GDPGR, GDP Growth Rate.

## Discussion

Breusch–Pagan Test and Hausman Test show that fine goodness of fits of regression models and F-statistics as the coefficient value is significant. Researchers have employed a general to specific strategy and discovered that all explanatory factors included in the reestimation are statistically significant. Even though Durbin–Watson (DW) Test confirmed that there is no second-order autocorrelation present, inconsistency is not the issue, as the models reveal a positive first-order autocorrelation and it does not imply that the estimates are inaccurate. The Hausman test result indicates that the fixed effect for testing hypotheses is the proper model. Fixed and random effects are not adopted in the model base pooled in LM test and F test. Separately remained the same for Bangladesh and India and rejected the pooled model at significant 5 and 1% levels based on F-Test and LM tests. Overall, the banking sector's profitability is not satisfactory enough for both countries (Table 4). Based on Table 6, comparatively profitability indicators such as ROA and ROE are not in a good position for an overall judgment, but the condition is more vulnerable for Bangladesh (ROA is 0.79% and ROE is 0.98%) than India. The model indicated that bank size (BS), EAR (1.98%), and LDR as drivers of profitability have a favorable impact on bank profitability in Bangladesh. Based on the table, the ROA for Bangladeshi and Indian private banks exhibited nearly identical changes. GDPGR is adversely associated with ROA, whereas ROE and BS, DAR, EAR, DER, and IR are positively connected with ROA. Finally, ROE is positively associated with EAR, LDR, and ROA, whereas it is adversely associated with BS, DAR, DTAR, DER, IR, and GDP growth rate. Sarwar et al. (2018), Budhathoki et al. (2020), and Horobet et al. (2021) all showed similar results in terms of the impact on production. According to some study, the size of a bank has a little bearing on the productivity or return on investment, i.e., Aladwan (2015), Adelopo et al. (2017), Fidanoski et al. (2017), and Tharu and Yogesh (2019). Banks' specific factors have a great impact on productivity. This study has proved it for both the countries. The bank's specific components of debt to equity ratio (DER), loan to deposit ratio (LDR), and equity to asset ratio (EAR), all have a significant impact on the profitability. The effect of some factors is positive, the effect of some factors is negative, and some elements have no effect. The point is, the effects of most factors are more or less proven. Several researches on the association between bank-specific characteristics and profitability (Alshatti, 2015; Kajola et al., 2018; Okere et al., 2018) showed comparable results to those of the current study. Chowdhury (2015), Uwuigbe et al. (2015), Ariyadasa et al. (2016), Aykut (2016), Muriithi et al. (2016), Opoku et al. (2016), Annor and Obeng (2017); and Kani (2017) obtained the opposite results, indicating that certain factors had little impact on profitability. (H1) The hypothesis that specific factors/variables (internal factors) of banks have no significant impact on profitability (ROA and ROE) of the banking sector in Bangladesh and India is hereby rejected null hypothesis 1 (H1), as evidenced by Tables 4, 7, 9, which show that specific factors have an impact on productivity. According to the findings, fixed effects models are preferred over pooled effects models, since the *P* values for both models are very low in F statistics, rejecting the null hypothesis (H2) at the 1% level of significance. DTAR and DER have a negative connotation with ROA, but EAR and LDR have a favorable connotation with ROA (Alharbi, 2017; Parvin et al., 2019). The effects of EAR and DTAR on ROA are statistically significant (Al-Jafari and Alchami, 2014; Ariyadasa et al., 2016) at the 1% level of significance. EAR, LDR, and DTAR all have a considerable favorable impact on ROE, implying that the higher the effect on productivity, the better. Several researchers (Onofrei et al., 2018; Cetin, 2019; Mosharrafa and Islam, 2021) have discovered similar results. The findings of Ariyadasa et al. (2016), Aydemir and Ovenc (2016), Tan et al. (2016), Alzoubi (2018); and Mohanty and Krishnankutty (2018) were also confirmed. Tables 8, 9 show that IR and GDPGR have a positive and significant impact on ROA, whereas GDPGR has a positive significant impact on ROE and IR has a negative significant impact on ROE. Aykut (2016), Tan and Anchor (2017), Tan and Floros (2018), Kajola et al. (2019), and Trusova et al. (2021) were among the researchers who confirmed the findings of the current investigation. We can rule out the null hypothesis (H2) based on the findings.

Microeconomic factors/variables (external factors) have no significant impact on profitability (ROA and ROE) of the banking sector in Bangladesh and India. The variables influencing the economics of Bangladesh and India are similar in most cases. Bangladesh is ahead in some indices while India is ahead in some indices. In the Banking system, India is more integrated, tidy, and successful than Bangladesh. However, Bangladesh is much more advanced than before. The results of the study showed that the effect of DAR and DTAR on ROA and ROE as a profitability indicator is positive significant in both cases. Especially for Bangladesh, BS, DAR, LDR, and GDP have a positive impact, but DTAR is to be found to be negative on ROA. For India, DAR, DTAR, and DER are found positive and significant but EAR, GDPGR, and IR are found to be negative and significant with ROA (Tables 10, 11). Thus, the analysis proves that there is a difference between Bangladesh and India (Tan, 2016; Brahmaiah, 2018) on the basis of banks' specific and macroeconomic factors. So, null hypothesis (H3) is hereby rejected.

## Conclusion

The objective of this paper was to analyze the impact of the determining factors on the profitability of Asian countries in special reference to Bangladesh and India. Considering bank's special factors that are called internal factors and macroeconomic factors that are called external factors found less or more significant impact on the probability of both factors. Bank's special factors are called internal factors and macroeconomic factors are called external factors. Internal factors are factors that are largely influenced by the management of a bank. This research analyzed the aggregate data of 20 banks in Bangladesh and 20 banks in India from 2010 to 2020 and examined independent variables such as ROA and ROE and dependent variables such as BS, DAR, DTAR, EAR, DER, LDR, IR, and GDPGR to determine the profitability of both countries' banks during the post-financial crisis. Through multiple regression analysis between ROA and bank's specific and macroeconomic variables and, ROE and bank's specific and macroeconomic variables the variability of determinants over different years was found. The analysis showed that the Bank size (BS), Debt to Asset Ratio (DAR), Inflation Rate (IR), and GDP Growth Rate (GDPGR) were found to have a positive impact and Deposit to Asset Ratio (DTAR) and Loan to Deposit Ratio o (LDR) were also found to be negative and significant on ROA for both countries. GDP Growth Rate (GDPGR), Bank size (BS), and EAR-Equity to Asset Ratio are positive and significant, whereas Deposit to Asset Ratio (DTAR), Inflation Rate (IR) and Debt to Equity Ratio (DER) are negative and significant for ROE. Due to fierce competition in the banking sector, the BS is not important for the bank's profitability of ROE and ROA to have a minor and puny significant impact. We see negative signs across all types of banks in terms of both the size of banks and the profitability relationship of Indian banks, but for Bangladeshi banks we found a positive significant impact on ROA and negative significant impact on ROE. Profitability analysis aids in comprehending the phenomenon of a company's healthy and sustainable financial situation. Profitability helps to justify the financial success and growth trends of a bank. As a result, it is crucial to investigate the factors that influence bank profitability. The result of this study is significant to policymakers, bankers, regulators, bank management, and other stakeholders. The findings will help bank management and shareholders to identify internal and external key factors for profit maximization, which in turn lead to stability at the bank level. This study included only two countries from South Asia and very few selective banks' specific and macroeconomic variables and did not include many others, which might affect the profitability of private banking sector in Bangladesh and India. Therefore, there is an opportunity to further investigate the profitability of the South Asian countries' banking sector. Lastly, the study suggests that future studies be guided by gathering more financial and economic data, as well as adopting cutting-edge research approaches. It seems important to expand a list of potential variables and include more countries, so that various data issues disappear and cannot be identified when running relevant diagnostics. This will help in conducting more accurate investigations and harmonizing the problem of profit making of banks.

## Data availability statement

The original contributions presented in the study are included in the article/supplementary material, further inquiries can be directed to the corresponding author/s.

## Author contributions

Data curation: MG, IH, and BD. Formal analysis, methodology, and conceptualization: DY and MG. Funding acquisition: DY and IH. Investigation: IH, BD, and AH. Project administration: MG and IH. Resources: AH, DY, and BD. Software: MG and BD. Supervision: AH and IH. Validation: YD and BD. Writing–original draft: MG. Writing–review and editing: IH, DY, BD, and MG. All authors have read and agreed to the published version of the manuscript.

## Funding

This research project was supported by the Foundation Project of National Natural Science Foundation of China (Grant Nos. 71962016 and 71962017) and received partial funding support from the Universitas Airlangga, Indonesia.

## Conflict of interest

The authors declare that the research was conducted in the absence of any commercial or financial relationships that could be construed as a potential conflict of interest.

## Publisher's note

All claims expressed in this article are solely those of the authors and do not necessarily represent those of their affiliated organizations, or those of the publisher, the editors and the reviewers. Any product that may be evaluated in this article, or claim that may be made by its manufacturer, is not guaranteed or endorsed by the publisher.
